# Annexin A1 (Ac2-26)-dependent Fpr2 receptor alleviates sepsis-induced acute kidney injury by inhibiting inflammation and apoptosis in vivo and in vitro

**DOI:** 10.1007/s00011-022-01640-9

**Published:** 2022-12-22

**Authors:** Yanlei Zheng, Yan Li, Shi Li, Ronghua Hu, Li Zhang

**Affiliations:** 1 Department of Critical Care Medicine, Hubei Cancer Hospital, Tongji Medical College, Huazhong University of Science and Technology, Wuhan, 430079 China; 2 Department of Critical Care Medicine, Xiangyang Central Hospital, Xiangyang, 440121 China

**Keywords:** Sepsis, ANXA1 (Ac2-26), Fpr2, AKI, Inflammation, Apoptosis

## Abstract

**Objectives:**

Excessive inflammatory responses and apoptosis are critical pathologies that contribute to sepsis-induced acute kidney injury (SI-AKI). Annexin A1 (ANXA1), a member of the calcium-dependent phospholipid-binding protein family, protects against SI-AKI through its anti-inflammatory and antiapoptotic effects, but the underlying mechanisms are still largely unknown.

**Methods:**

In vivo, SI-AKI mouse models were established via caecal ligation and puncture (CLP) and were then treated with the Ac2-26 peptide of ANXA1 (ANXA1 (Ac2-26)), WRW4 (Fpr2 antagonist) or both. In vitro, HK-2 cells were induced by lipopolysaccharide (LPS) and then treated with ANXA1 (Ac2-26), Fpr2–siRNA or both.

**Results:**

In the present study, we found that the expression levels of ANXA1 were decreased, and the expression levels of TNF-α, IL-1β, IL-6, cleaved caspase-3, cleaved caspase-8 and Bax were significantly increased, accompanied by marked kidney tissue apoptosis in vivo. Moreover, we observed that ANXA1 (Ac2-26) significantly reduced the levels of TNF-α, IL-1β and IL-6 and cleaved caspase-3, cleaved caspase-8, FADD and Bax and inhibited apoptosis in kidney tissue and HK-2 cells, accompanied by pathological damage to kidney tissue. Seven-day survival, kidney function and cell viability were significantly improved in vivo and in vitro, respectively. Furthermore, the administration of ANXA1 (Ac2-26) inhibited the CLP- or LPS-induced phosphorylation of PI3K and AKT and downregulated the level of NF-κB in vivo and in vitro. Moreover, our data demonstrate that blocking the Fpr2 receptor by the administration of WRW4 or Fpr2–siRNA reversed the abovementioned regulatory role of ANXA1, accompanied by enhanced phosphorylation of PI3K and AKT and upregulation of the level of NF-κB in vivo and in vitro.

**Conclusions:**

Taken together, this study provides evidence that the protective effect of ANXA1 (Ac2-26) on SI-AKI largely depends on the negative regulation of inflammation and apoptosis via the Fpr2 receptor.

## Introduction

Currently, sepsis remains one of the leading clinical conditions in intensive care units (ICUs) and causes a substantial economic burden worldwide [1–3]. The kidney is one of the most vulnerable organs and is associated with severe complications in sepsis [4], which is an important cause of acute kidney injury (AKI) in ICU patients [5, 6]. Sepsis-induced acute kidney injury (SI-AKI) is associated with a poor prognosis and high mortality in critically ill patients [7, 8]. Related studies have reported that the mortality rate of patients with SI-AKI is significantly higher than that of patients with sepsis without AKI [9]. With advances in clinical treatment, such as fluid resuscitation and kidney replacement therapy, these treatments may improve survival, but the mortality rate of patients with AKI is still as high as 30% [10]. There is still no specific treatment for SI-AKI, and its mechanisms are not fully understood.

Numerous studies have suggested that the pathogenesis of SI-AKI is due to intrarenal haemodynamic abnormalities, the inflammatory response, immune cell infiltration and renal tubular cell apoptosis [8, 11–13]. Further research has found that the inflammatory response and apoptosis in renal tissue are closely related to the occurrence of SI-AKI [14, 15]. When the host has a severe bacterial infection, a large increase in lipopolysaccharide (LPS) in the serum leads to significant increases in proinflammatory factors, which eventually induce tissue damage and organ failure, also known as sepsis [16]. In addition, studies have reported that fish oil attenuates septic acute kidney injury induced by caecal ligation and puncture (CLP) by negatively regulating inflammation, oxidative stress and apoptosis [17]. Currently, LPS and CLP are widely used in sepsis and septic kidney injury research.

Annexin A1 (ANXA1) is a member of the calcium-dependent phospholipid-binding protein family of annexins and is a 37 kDa protein composed of 346 amino acids that is expressed in multiple organs, such as the heart, lung, brain, and kidney [18, 19]. It is currently believed that ANXA1 is involved in various biological regulatory pathways through its N-terminal peptide of Ac2-26, such as the regulation of the inflammatory response, cell signalling, cell differentiation, and apoptosis [20, 21]. Related studies have shown that ANXA1 (Ac2-26) protects against myocardial injury in rats with sepsis by negatively regulating myocardial apoptosis [22]. Wu et al. found that ANXA1 (Ac2-26) protects against kidney injury by inhibiting inflammatory responses in diabetic nephropathy [23]. Further studies reported that ANXA1 (Ac2-26) could negatively regulate the inflammatory response and apoptosis through the Fpr2 receptor [24–26]. The Fpr2 receptor is a member of the G protein-coupled receptors, comprising Fpr1, Fpr2 (orthologue in mouse), and Fpr3, which are involved in host defence and regulation of inflammation [27]. Fpr2 is expressed in myeloid cells, dendritic cells, macrophages and endothelial cells [28, 29]. Moreover, some studies have reported that FPR2 is expressed in the central nervous system [30], kidney tissues [31] and lung tissues [32] and that FPR2 is rapidly upregulated after inflammatory injury [33]. Senchenkova et al. and Kreutter et al. found that a selective Fpr2 receptor antagonist (WRW4) blocks the anti-inflammatory and anti-apoptotic effects of ANXA1 in mice [34, 35]. However, the functional role of ANXA1 (Ac2-26) in SI-AKI and whether ANXA1 (Ac2-26) can regulate inflammation and apoptosis in SI-AKI through the Fpr2 receptor remain largely unknown.

In this study, we hypothesized that ANXA1 (Ac2-26) has a protective effect in SI-AKI. To verify this hypothesis, we established mouse and HK-2 cell experimental models by CLP and LPS stimulation, respectively, to investigate the effects of ANXA1 (Ac2-26) in SI-AKI in vivo and in vitro.

## Materials and methods

### Materials

ANXA1 (Ac2-26) (acetyl-AMVSEFLKQAWFIEEQEYVQTVK) was purchased from Hangzhou Angtai Biotechnology Co. Ltd. (China). The Cre and BUN ELISA kits were obtained from Nanjing Jiancheng Bioengineering Institute (China). The p-PI3K, PI3K, p-AKT, AKT, NF-κB, cleaved caspase-3, cleaved caspase-8 and FADD primary antibodies were obtained from Abcam (USA). The Bcl-2 and Bax primary antibodies were purchased from Cell Signaling Technology, Inc. (Danvers, MA, USA). The TUNEL kit was an In Situ Cell Death Detection Kit and was purchased from Roche (USA). The CCK-8 kit was obtained from Dojindo Molecular Technologies (Gaithersburg, MD, USA). The Annexin V-FITC flow cytometry apoptosis kit was purchased from Tianjin Sungene Biotech Co., Ltd. (China).

### Animals and modelling

Male wild-type C57BL/6 mice (6–8 weeks, weighing 20–30 g) were obtained from the Center of Experimental Animals of Wuhan University in China. The mice were maintained in a standard environment (12-h light/dark cycle, temperature of 25 °C, and humidity of 50%) with free access to food and water for 1 week before the experiments. The mouse experiments were performed in the animal laboratory of Hubei Provincial Cancer Hospital. The experimental model of sepsis was established by CLP [36]. The mice were anaesthetized by 1% sodium pentobarbital (50 mg/kg) and then sacrificed by cervical dislocation before the procurement of their tissue specimens. The mice were induced with ANXA1 (Ac2-26) (1 mg/kg), WRW4 (1.8 mg/kg), or both, and the effect of ANXA1 (Ac2-26) on CLP-induced AKI was determined in vivo.

### HK-2 Cells

HK-2 cells were cultured in DMEM (Gibco, Thermo Fisher Scientific, Waltham, MA, USA) containing 10% foetal bovine serum (FBS, Gibco, Thermo Fisher Scientific, Waltham, MA, USA). HK-2 cells were induced with LPS (10 μg/mL), ANXA1 (Ac2-26) (0.5 μmol/L), Fpr2-siRNA, or all three, and the effect of ANXA1 (Ac2-26) on LPS-induced HK-2 cells was determined in vitro.

### HK-2 cell viability assay

The viability of HK-2 cells was determined by a cell counting kit 8 (CCK-8) assay according to the manufacturer’s instructions. The viability of HK-2 cells was calculated as follows: (experimental group/control group) × 100.

### Detection of Cre, BUN and inflammatory cytokine levels by ELISA

The levels of Cre, BUN and inflammatory cytokines were detected by ELISA kits according to the manufacturer’s instructions.

### Reverse transcription–quantitative polymerase chain reaction (RT–qPCR)

The mRNA expression levels of TNF-α, IL-1β and IL-6 in kidney tissue were measured by RT–qPCR. First, total RNA was extracted by TRIzol reagent (Invitrogen Life Technologies, CA, USA). Then, cDNA was synthesized from the RNA with PrimeScript™ RT Master Mix (Takara, Japan) for quantitative RT–PCR. The sequences of the sample primers are shown in Table 1.

**Table 1:** Primer sequences used for quantitative RT–PCR

Genes	Forward primer sequence (5′ → 3′)	Reverse forward primer sequence (5′ → 3′)
TNF-α	CACCACGCTCTTCTGTCTACTG	GCTACGGGCTTGTCACTCG
IL-1β	AGTTGACCGACCCCAAAAG	AGCTGGATGCTCTCATCAGG
IL-6	GACAAAGCCGAGTCATTCAGAG	GTCTTGGTCCTTAGCCACTCC
GAPDH	GCCAAGGTCATCCATGACAAC	GTGGATGCAGGGATGATGTTC

### Western blot analysis

The protein levels of p-PI3K, PI3K, p-AKT, AKT, NF-κB, cleaved caspase-3, cleaved caspase-8, FADD, Bcl-2 and Bax in kidney tissue and HK-2 cells were measured by western blot analysis. First, the proteins were extracted with RIPA lysis buffer from kidney tissue, and the concentration was determined via a bicinchoninic acid (BCA) assay. Each sample was electrophoresed on an SDS–polyacrylamide gel and transferred to PVDF membranes. The PVDF membranes were blocked with PBS containing 5% skim milk and incubated with primary antibodies (anti-MyD88, 1:1000; anti-p-AKT, 1:1000; anti-AKT, 1:1000; anti-p-PI3K, 1:1000; anti-PI3K, 1:1000; anti-NF-κB, 1:1000; anti-cleaved caspase3, 1:800; anti-caspase8, 1:800; anti-FADD, 1:800; anti-Bcl-2, 1:800; and anti-Bax, 1:1000) in a shaker at 4 °C overnight. Next, the membranes were washed with TBST and then incubated with secondary antibodies (LI-COR, Lincoln, NE, USA; 1:5000 dilution) for 1 h at room temperature. Finally, the PVDF membranes were visualized by a LI-COR Odyssey Infrared Imaging System (LI-COR Biosciences Inc., USA).

### Haematoxylin and eosin staining of kidney tissue

The kidney tissue was collected and fixed with a 10% formaldehyde solution for 24 h at room temperature, embedded in paraffin and cut into 3-µm slices. Then, the sections were stained with haematoxylin and eosin (HE) and assessed to determine morphological changes and damage under a light microscope. The degree of pathological injury was scored based on oedema, tubule dilatation, etc., according to a previously reported scoring system [37].

### Transmission electron microscopy analysis of kidney tissue

The kidney tissue was harvested and fixed with 2.5% formaldehyde in PBS solution. Next, the tissue was postfixed with OsO4 for 1 h at room temperature, washed with PBS, dehydrated in ethanol, embedded in epoxy resin and cut into ultrathin sections. The tissue sections were observed under an H-7100 transmission electron microscope (TEM) (Hitachi Co., Japan) operating at 75 kV.

### Terminal dUTP nick end-labelling (TUNEL) assay

Kidney tissue was collected and fixed with 10% formaldehyde, embedded in paraffin and sliced into sections. The tissue sections were stained with a TUNEL kit according to the manufacturer’s instructions. The sections were stained with 4′,6-diamidino-2-phenylindole (DAPI) for 15 min, washed with PBS and observed under a fluorescence microscope. The apoptotic index was calculated as follows: (AI) = positive cells/total cells × 100.

### Flow cytometry analysis of HK-2 cell apoptosis

The apoptosis rate of HK-2 cells was determined by an Annexin V-FITC flow cytometry apoptosis kit. Briefly, 200 μL binding buffer containing 10 μL Annexin V and 5 μl PI was added to the samples for 1 h in the dark. The stained cells were analysed using a FACSCalibur™ flow cytometer (BD Biosciences, San Jose, CA, USA) and Cell-Quest software.

### Statistical analysis

SPSS version 20.0 statistical software (SPSS, Inc., USA) was used for the statistical analysis. All data are presented as the means ± standard deviations (SD). Student's t test was used to compare the differences between two groups. One-way analysis of variance (ANOVA), followed by Bonferroni’s post-hoc analysis, was performed for comparisons of the differences among more than two groups. *P* values < 0.05 were considered statistically significant.

## Results

### CLP induction significantly downregulated the level of ANXA1 and increased the levels of inflammatory cytokines and apoptotic proteins, accompanied by apoptosis in the kidney, in CLP-treated mice

**Fig. 1:**
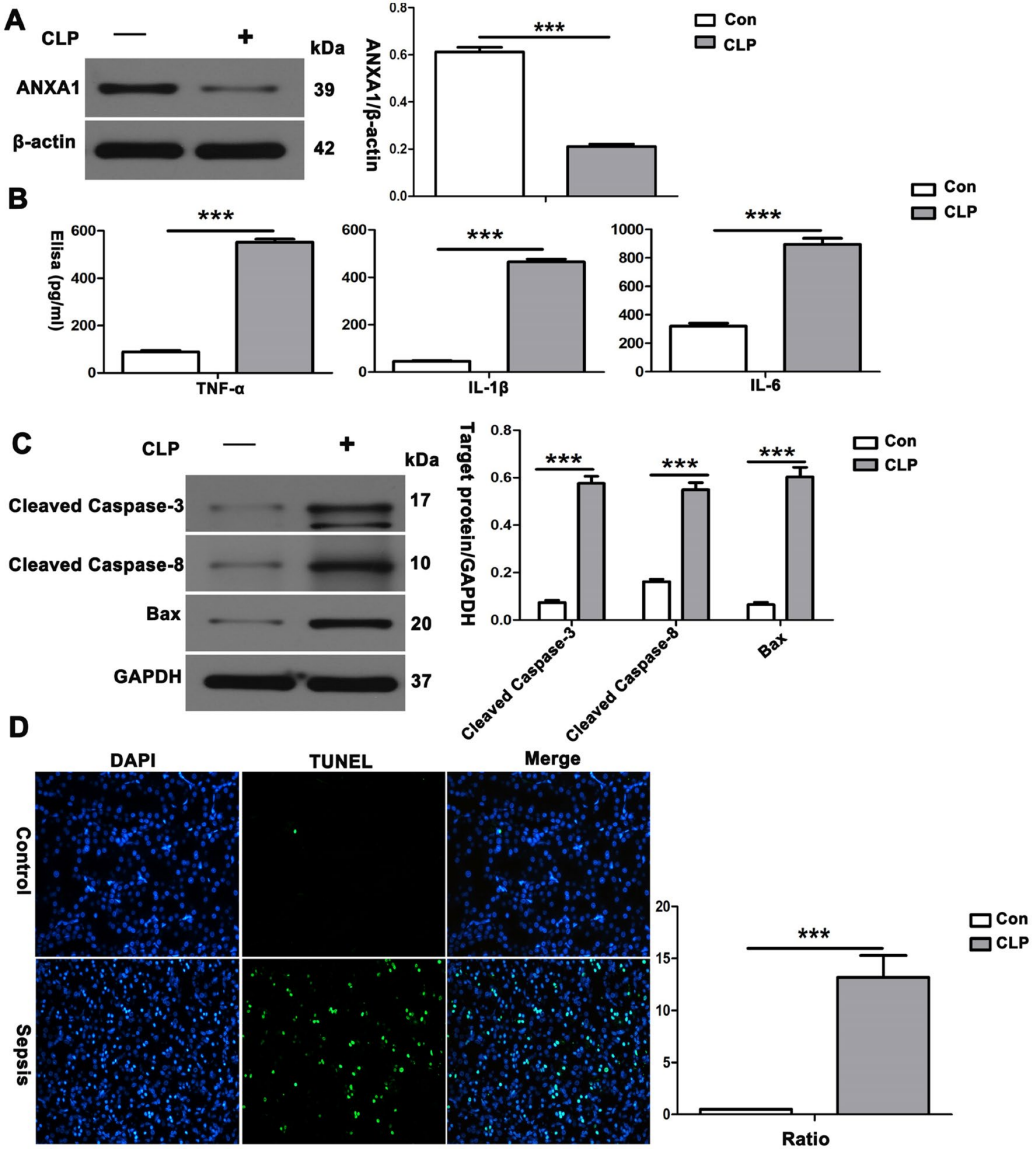
Reduction in ANXA1 produced a significant increase in inflammatory cytokines, proapoptotic proteins and apoptosis in kidney tissue in mice. **A** Representative western blots and quantification results of ANXA1 after CLP for 24 h. β-Actin was used as a loading control for the total protein. **B** TNF-α, IL-1β and IL-6 levels in the serum of mice were determined by ELISA after CLP for 24 h. **C** Representative western blots and quantification results of cleaved caspase-3, cleaved caspase-8 and Bax after CLP for 24 h. GAPDH was used as a loading control for the total protein. **D** Renal tissue apoptosis was determined by TUNEL after CLP for 24 h. Note: Con: control group, CLP: CLP group. The data are represented as the mean ± SEM. The data were independently replicated in triplicate. ***P < 0.001

To explore the role of ANXA1 in kidney tissue inflammation and apoptosis in vivo, we constructed a mouse AKI model by CLP [38]. First, we found that the level of endogenous ANXA1 in the kidney tissue in the sepsis group was significantly lower than that in the controls (Fig. 1A). Consistently, the ELISAs of TNF-α, IL-1β and IL-6 showed that the serum TNF-α, IL-1β and IL-6 levels in the sepsis group were higher than those in the control group (Fig. 1B). Next, kidney tissue from the CLP group was collected for western blot analysis. Our data showed that the proapoptotic proteins cleaved caspase-3, cleaved caspase-8 and Bax were dramatically increased in CLP-treated mice compared with control mice (Fig. 1C). In addition, a TUNEL assay of apoptosis in kidney tissue showed apoptosis in kidney tissue in the CLP group compared with the control group (Fig. 1D). Taken together, these results suggest that elevated levels of inflammatory cytokines and apoptotic proteins and apoptosis in renal tissue are associated with the downregulation of ANXA1 expression levels in SI-AKI induced by CLP in mice.

### ANXA1 (Ac2-26) reduces the levels of inflammatory cytokines and pathological damage in kidney tissue through the Fpr2 receptor in CLP-induced mice

**Fig. 2:**
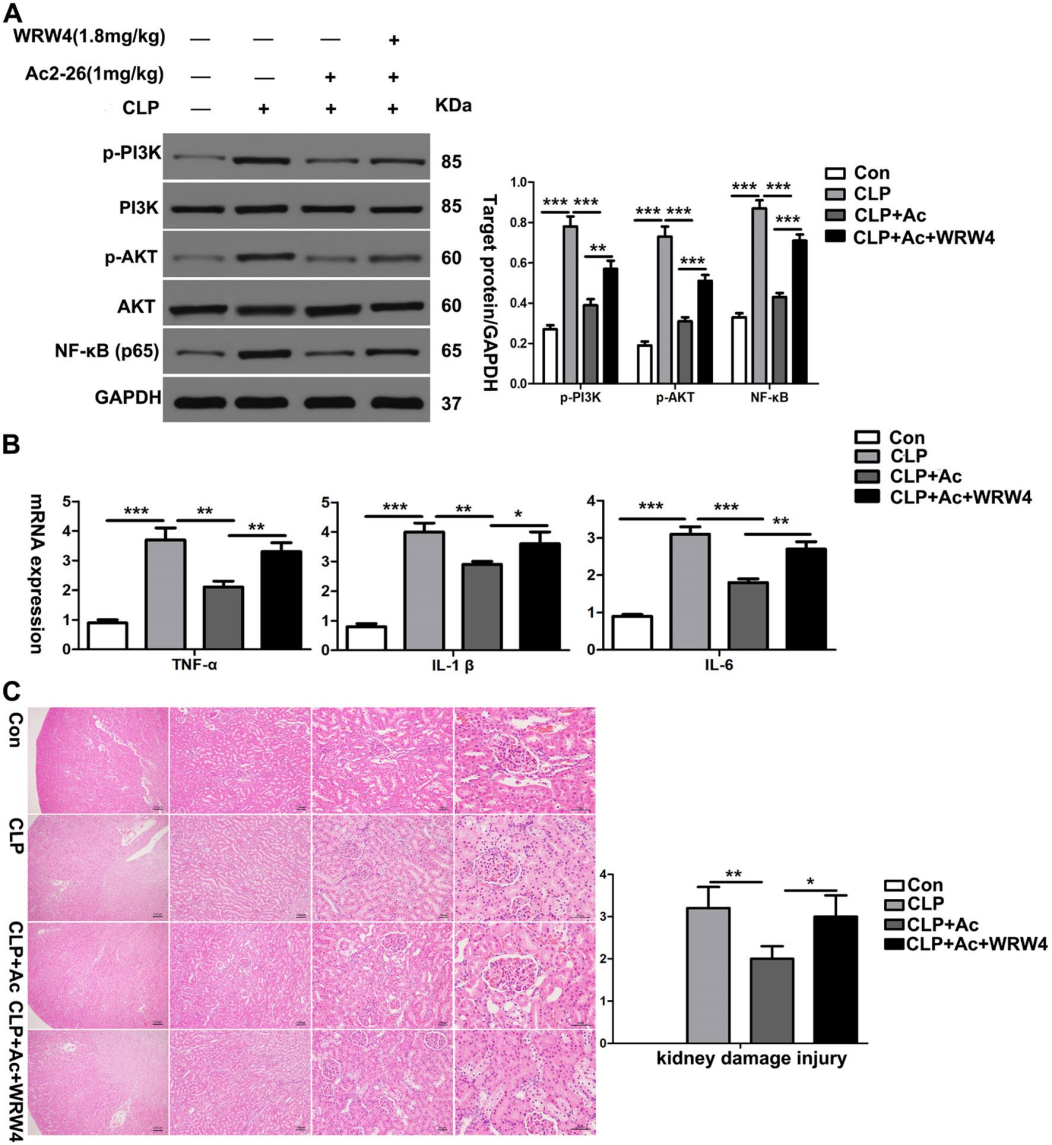
ANXA1 (Ac2-26) decreased the levels of inflammatory cytokines and pathological damage in kidney tissue from CLP-induced mice. **A**–**C** At 24 h after the CLP operation, the inflammatory cytokine levels were detected by a western blot analysis and RT–PCR. The kidney histopathological change was detected by HE staining (× 40, × 100, × 200 and × 400). Note: Ac: ANXA1 (Ac2-26) (1 mg/kg), WRW4 (1.8 mg/kg); Con: control group; CLP: CLP group; CLP + Ac: CLP + Ac2-26 group; CLP + Ac + WRW4: CLP + Ac2-26 + WRW4 group. The data are represented as the mean ± SEM. The data were independently replicated in triplicate. *P < 0.05, **P < 0.01, ***P < 0.001

Next, we determined whether the regulatory effects of ANXA1 (Ac2-26) on inflammation and kidney tissue pathological damage are mediated through the Fpr2 receptor and explored its downstream signalling pathways. We used WRW4 to block the Fpr2 receptor in CLP-induced mice. The results showed that CLP significantly increased the levels of inflammatory cytokines (TNF-α, IL-1β and IL-6), enhanced the phosphorylation of PI3K and AKT, and upregulated the level of NF-κB in the kidney tissue of the mice. In contrast, ANXA1 (Ac2-26) obviously decreased the levels of inflammatory cytokines (TNF-α, IL-1β and IL-6), inhibited the phosphorylation of PI3K and AKT, and downregulated the level of NF-κB in the kidney tissue of the mice (Fig. 2A, B). Moreover, treatment with ANXA1 (Ac2-26) significantly improved histopathological injury to the kidney (Fig. 2C). In addition, previous studies reported that ANXA1 (Ac2-26) inhibits inflammatory responses through Fpr2 in LPS-induced uveitis in rats [39]. The data showed that WRW4 reversed the negative regulation of inflammatory cytokines and kidney tissue injury by ANXA1 while significantly promoting the phosphorylation of PI3K and AKT and increasing the levels of NF-κB in CLP-induced mice (Fig. 2A–C). In summary, these results suggest that ANXA1 (Ac2-26) inhibits inflammation and kidney tissue damage by the Fpr2 receptor in CLP-induced mice.

### ANXA1 (Ac2-26) reduces the level of apoptosis-related proteins in kidney tissue via the Fpr2 receptor in CLP-induced mice

**Fig. 3:**
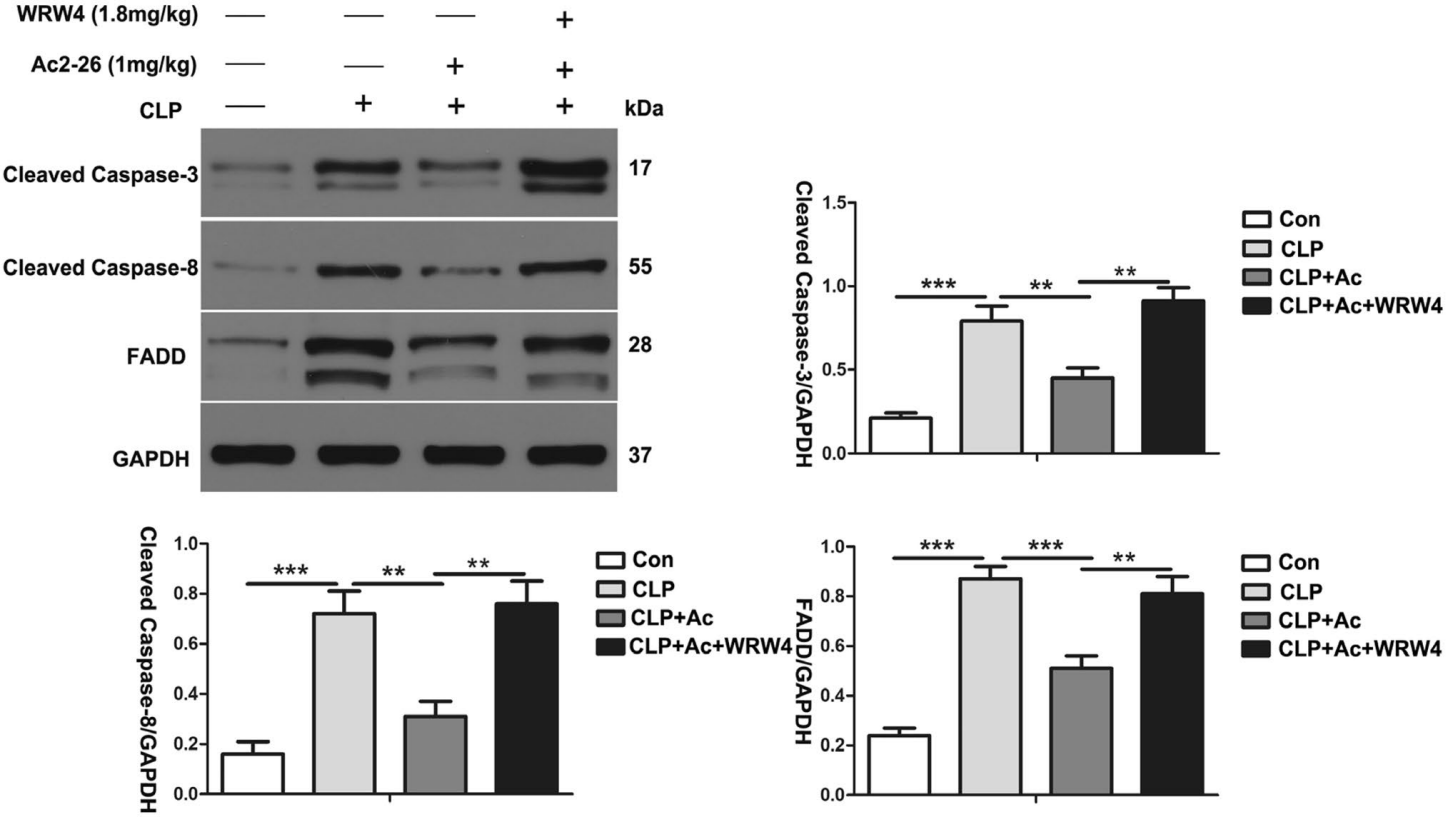
ANXA1 (Ac2-26) decreased the levels of apoptosis-related proteins in the kidney tissue of CLP-induced mice. The apoptosis-related protein levels were detected by a western blot analysis. Representative western blots and quantification results of cleaved caspase-3, cleaved caspase-8 and FADD after CLP for 24 h. GAPDH was used as a loading control for the total protein. Note: Ac: ANXA1 (Ac2-26) (1 mg/kg), WRW4 (1.8 mg/kg); Con: control group; CLP: CLP group; CLP + Ac: CLP + Ac2-26 group; CLP + Ac + WRW4: CLP + Ac2-26 + WRW4 group. The data are represented as the mean ± SEM. The data were independently replicated in triplicate. **P < 0.01, ***P < 0.001

To further investigate the effect of ANXA1 (Ac2-26) on the levels of apoptosis-related proteins in kidney tissue from CLP-treated mice, we performed western blot analysis. Our data show that CLP markedly upregulated the expression of cleaved caspase-3, cleaved caspase-8 and FADD in the kidney tissue from the mice. In contrast, ANXA1 (Ac2-26) obviously downregulated the CLP-induced increases in the expression of cleaved caspase-3, cleaved caspase-8 and FADD in the kidney tissue from the mice (Fig. 3). It has been reported that ANXA1 inhibits apoptosis in beta cells through Fpr2 in islets [35]. Interestingly, we found that the administration of the Fpr2 receptor blocker WRW4 reversed the negative regulation of apoptosis-related proteins by ANXA1 (Ac2-26) in kidney tissue from CLP-induced mice (Fig. 3). Taken together, these data indicate that ANXA1 (Ac2-26) inhibits the expression levels of apoptosis proteins in kidney tissue via the Fpr2 receptor in CLP-induced mice.

### ANXA1 (Ac2-26) inhibits apoptosis in kidney tissue via the Fpr2 receptor in CLP-induced mice

**Fig. 4:**
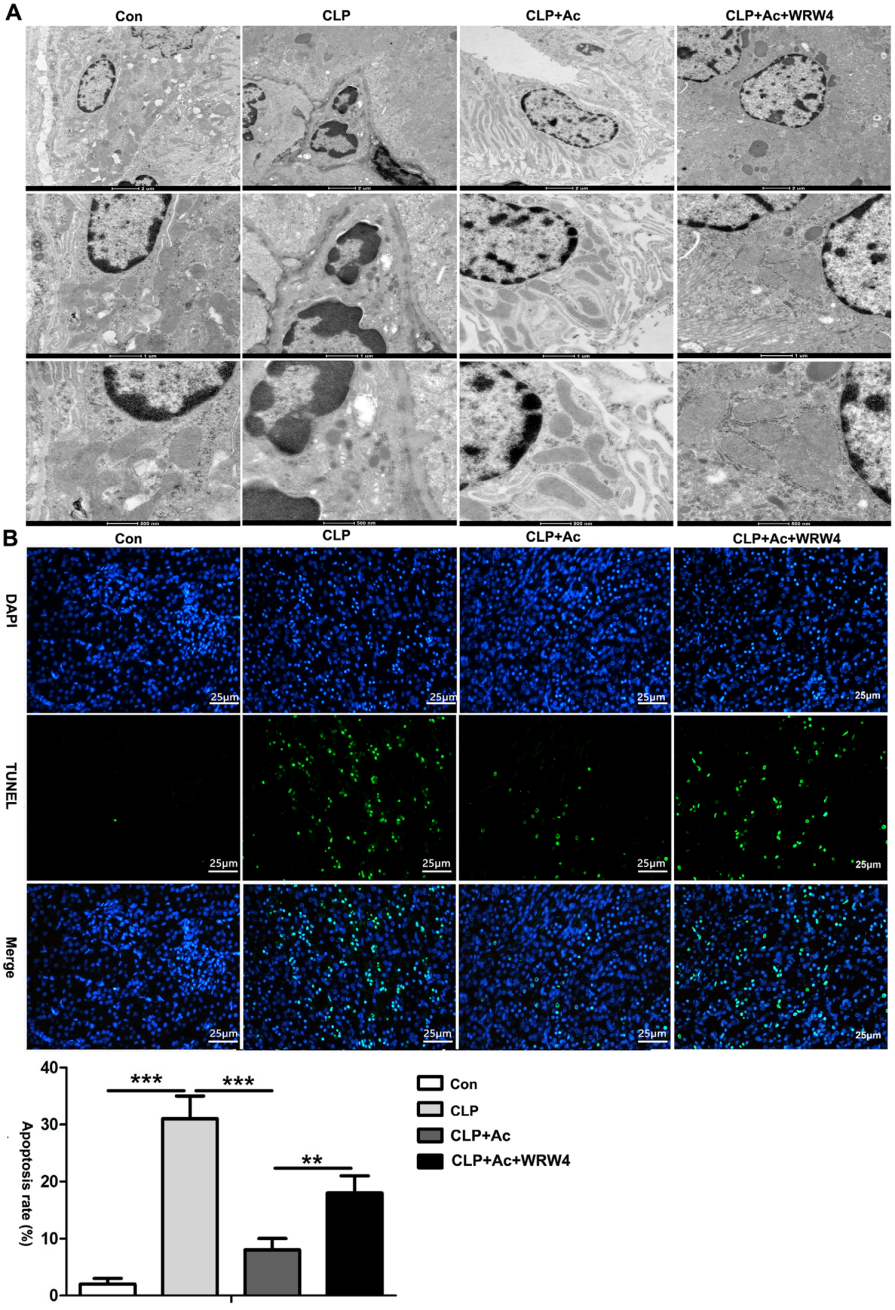
ANXA1 (Ac2-26) inhibits apoptosis in kidney tissue by Fpr2 receptor in CLP-mice. **A**, **B** At 24 h after the CLP operation, apoptosis in kidney tissue was measured by TEM and TUNEL. **A** (× 2900, × 6800 and × 13,000); **B** TUNEL-positive cells showed green fluorescence: apoptotic cells (× 400). Note: Ac: ANXA1 (Ac2-26) (1 mg/kg), WRW4 (1.8 mg/kg); Con: control group; CLP: CLP group; CLP + Ac: CLP + Ac2-26 group; CLP + Ac + WRW4: CLP + Ac2-26 + WRW4 group. The data are represented as the mean ± SEM. The data were independently replicated in triplicate. *P < 0.05, **P < 0.01, ***P < 0.001

To further explore the effect of ANXA1 on CLP-induced kidney tissue apoptosis, TEM and TUNEL assays were performed in mice. The TEM images revealed apoptotic bodies, mitochondrial swelling, partial spine fragmentation, vacuoles and deformation in the kidneys from the CLP-treated mice (Fig. 4A). Furthermore, the TUNEL staining results showed that the number of apoptotic cells was significantly increased in the kidneys of the CLP-treated mice (Fig. 4B). In contrast, ANXA1 (Ac2-26) obviously markedly reduced apoptosis in kidney tissue from CLP-treated mice (Fig. 4A, B). To further verify whether ANXA1 regulates kidney apoptosis through the Fpr2 receptor, treatment with WRW4 markedly reversed the protective effect of ANXA1 (Ac2-26) on apoptosis in the kidneys from CLP-induced mice (Fig. 4A, B). The above results suggest that ANXA1 inhibits CLP-induced kidney apoptosis through the Fpr2 receptor.

### ANXA1 (Ac2-26) attenuates LPS-induced inflammatory cytokine production in HK-2 cells via the Fpr2 receptor

**Fig. 5:**
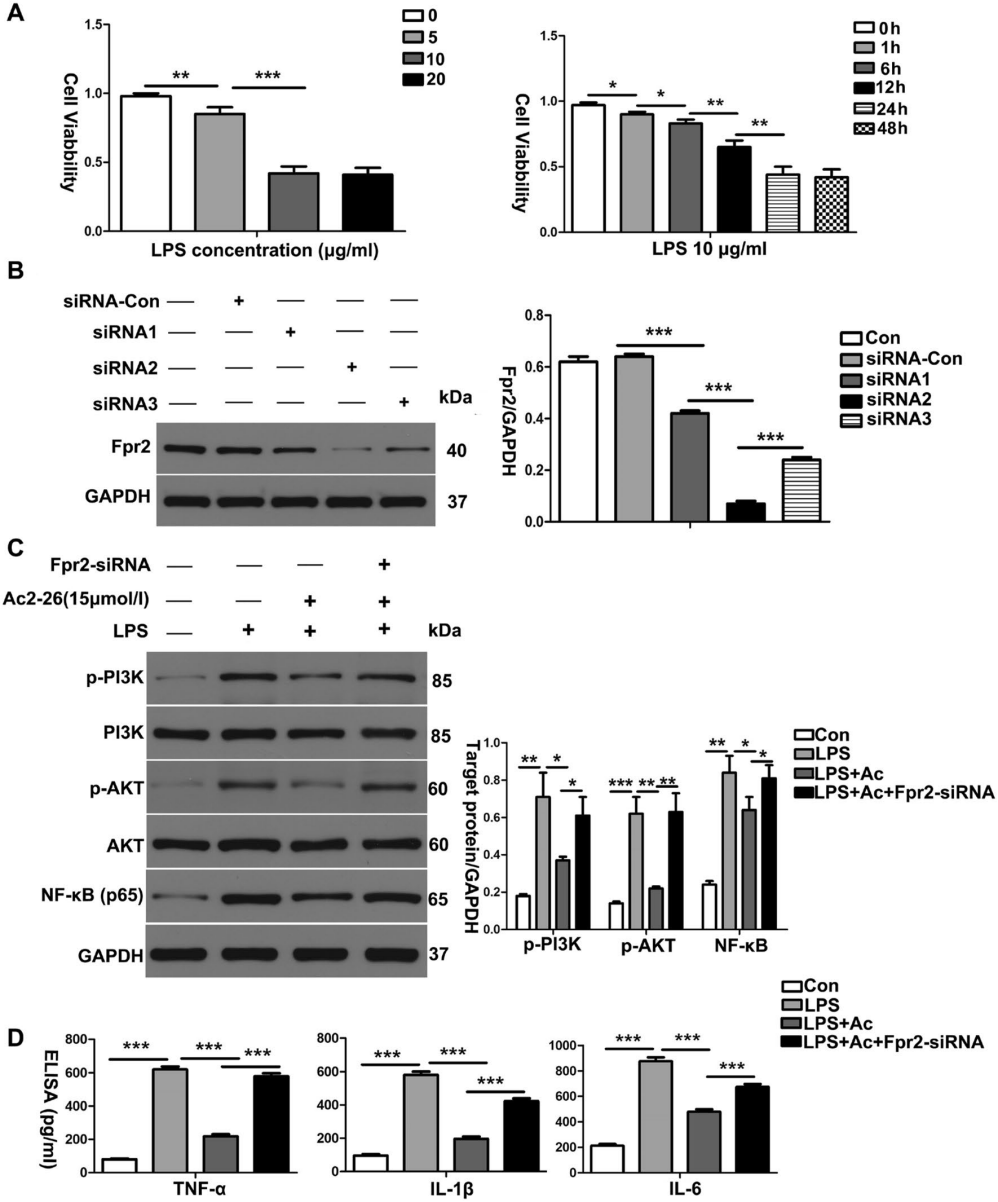
ANXA1 (Ac2-26) inhibits the production of inflammatory cytokines in LPS-induced HK-2 cells. **A** The effects of different concentrations of LPS and LPS (10 µg/ml) at different times on the activity of HK-2 cells were detected by CCK-8; **B**, **C** effect of Fpr2-siRNA-transfected HK-cells on Fpr2 and the effect of ANXA1 on the expression levels of inflammatory cytokines in HK-2 cells were detected by a western blot analysis; **D** TNF-α, IL-1β and IL-6 levels in culture supernatants were determined by ELISA after LPS (10 µg/ml) for 24 h. Note: LPS (10 μg/mL), Ac: ANXA1 (Ac2-26) (0.5 μmol/L); Con: control group, LPS: LPS group, LPS + Ac: LPS + Ac2-26 group, LPS + Ac + Fpr2-siRNA: LPS + Ac2-26 + Fpr2-siRNA. The data are represented as the mean ± SEM. The data were independently replicated in triplicate. *P < 0.05, **P < 0.01, ***P < 0.001

To further study the effects of ANXA1 on the levels of inflammatory cytokines at the cellular level during sepsis, we constructed an experimental cell model by inducing HK-2 cells with LPS (10 µg/ml) for 24 h. The levels of inflammatory cytokines were measured by western blot analysis and ELISA. Our western blot analysis and ELISA results showed that the LPS-induced production of TNF-α, IL-1β and IL-6 was significantly increased, while LPS enhanced the phosphorylation of PI3K and AKT and upregulated the level of NF-κB in HK-2 cells (Fig. 5C, D). Interestingly, treatment with ANXA1 (Ac2-26) markedly attenuated the production of TNF-α, IL-1β and IL-6, inhibited the phosphorylation of PI3K and AKT, and downregulated the level of NF-κB in LPS-treated HK-2 cells (Fig. 5C, D). Furthermore, we observed that the administration of Fpr2-siRNA reversed the negative regulation by ANXA1 of the above molecules induced by LPS in HK-2 cells (Fig. 5C, D). These data suggest that ANXA1 (Ac2-26) attenuates inflammation via the Fpr2 receptor in LPS-treated HK-2 cells.

### ANXA1 (Ac2-26) reduces LPS-induced apoptosis-related protein production in HK-2 cells via the Fpr2 receptor

**Fig. 6:**
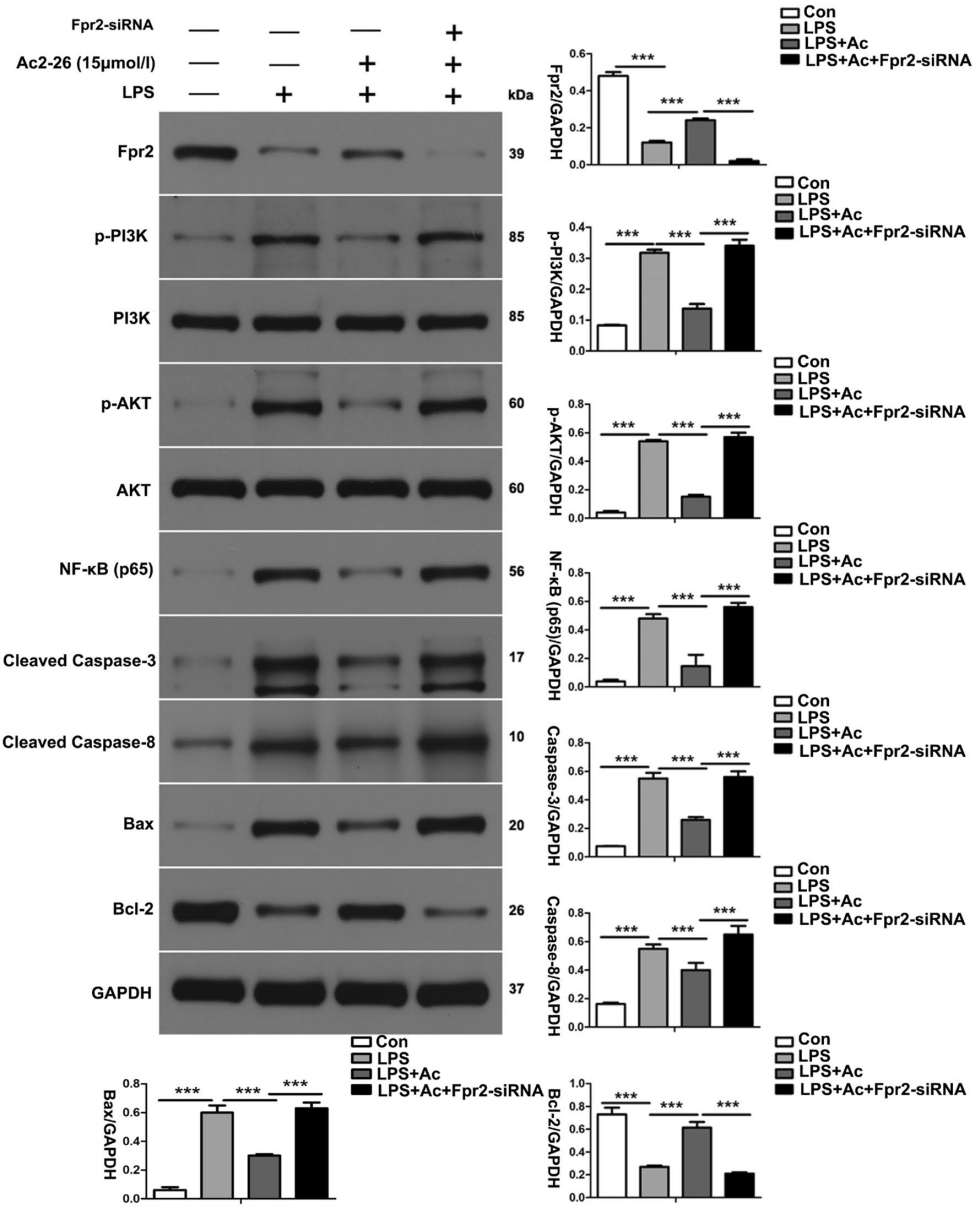
ANXA1 (Ac2-26) reduces LPS-induced apoptosis-related protein expression in HK-2 cells via the Fpr2 receptor. Apoptosis-related protein levels were detected by a western blot analysis. Representative western blots and quantification results of pro-apoptosis (cleaved caspase-3, cleaved caspase-8 and Bax) and anti-apoptosis (Bcl-2) after LPS (10 µg/ml) for 24 h. GAPDH was used as a loading control for the total protein. Note: LPS (10 μg/mL), Ac: ANXA1 (Ac2-26) (0.5 μmol/L); Con: control group, LPS: LPS group, LPS + Ac: LPS + Ac2-26 group, LPS + Ac + Fpr2-siRNA: LPS + Ac2-26 + Fpr2-siRNA. The data are represented as the mean ± SEM. The data were independently replicated in triplicate. ***P < 0.001

To further investigate the effect of ANXA1 (Ac2-26), the expression levels of apoptosis-related proteins in LPS-induced HK-2 cells were measured by western blot analysis. Our western blot analysis showed that the LPS-induced expression levels of cleaved caspase-3, cleaved caspase-8, and Bax were significantly upregulated and that Fpr2 and Bcl-2 were significantly downregulated, while LPS promoted the phosphorylation of PI3K and AKT and upregulated the level of NF-κB in HK-2 cells (Fig. 6). In contrast, ANXA1 (Ac2-26) obviously downregulated the LPS-induced expression of cleaved caspase-3, cleaved caspase-8, and Bax, obviously upregulated the LPS-induced expression of Fpr2 and Bcl-2, inhibited the phosphorylation of PI3K and AKT and downregulated the level of NF-κB in HK-2 cells (Fig. 6). Interestingly, we also found that the transfection of Fpr2-siRNA could reverse the negative regulation by ANXA1 (Ac2-26) in LPS-induced HK-2 cells (Fig. 6). These results indicate that ANXA1 downregulated the expression levels of proapoptotic proteins and upregulated the expression of antiapoptotic proteins by the Fpr2 receptor in LPS-treated HK-2 cells.

### ANXA1 (Ac2-26) inhibits apoptosis in LPS-treated HK-2 cells via the Fpr2 receptor

**Fig. 7:**
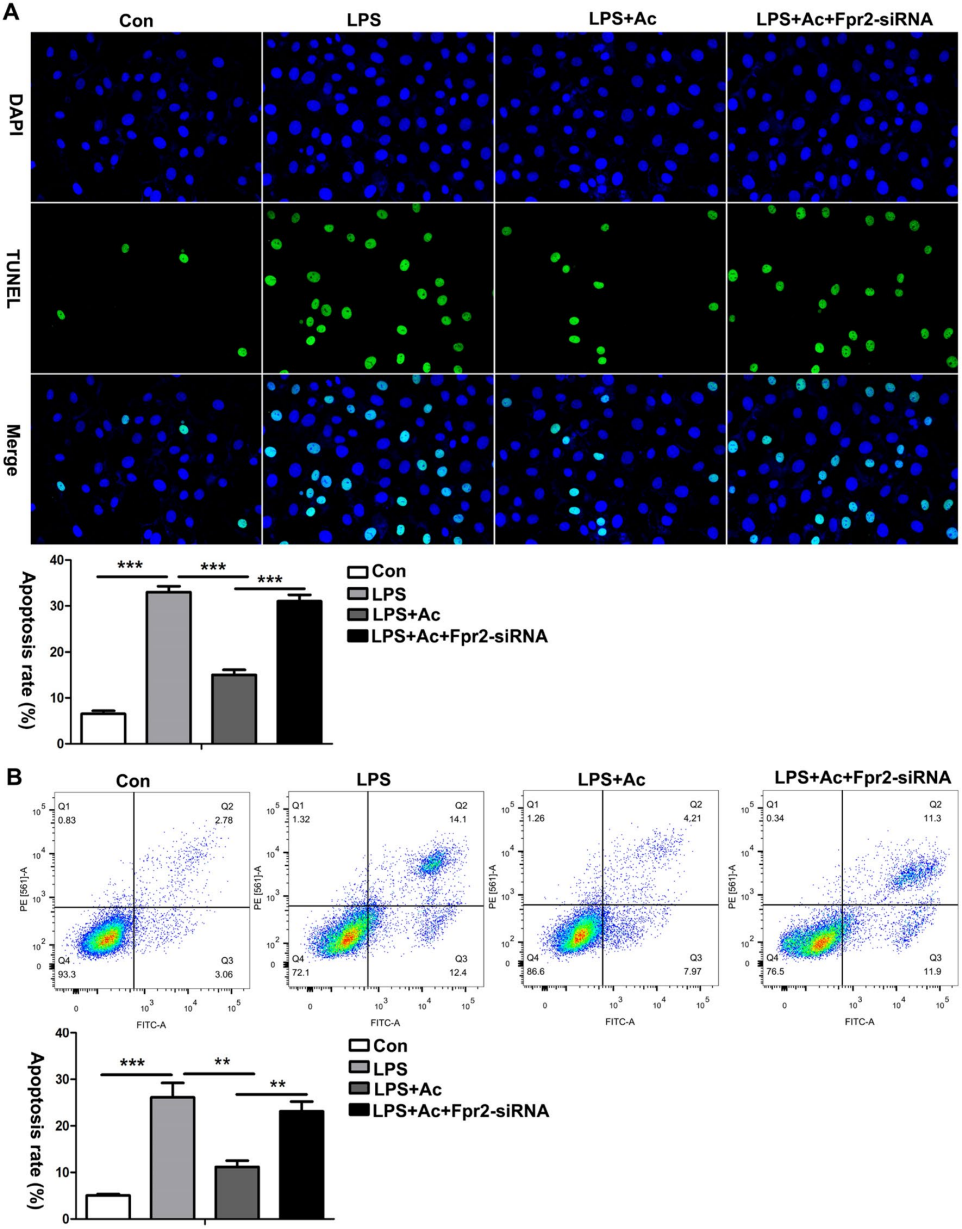
ANXA1 (Ac2-26) inhibits LPS-induced apoptosis in HK-2 cells via Fpr2 receptor. **A**, **B** LPS-induced apoptosis detected by TUNEL and flow cytometry in HK-2 cells after LPS (10 µg/ml) for 24 h. Note: TUNEL-positive cells showed green fluorescence: apoptotic cells (× 400). Note: LPS (10 μg/mL), Ac: ANXA1 (Ac2-26) (0.5 μmol/L); Con: control group; LPS: LPS group; LPS + Ac: LPS + Ac2-26 group; LPS + Ac + Frp2-siRNA: LPS + Ac2-26 + Fpr2-siRNA group. The data are represented as the mean ± SEM. The data were independently replicated in triplicate. **P < 0.01, ***P < 0.001

To further study the effect of ANXA1 on the apoptosis of LPS-treated HK-2 cells, TUNEL staining and flow cytometry were performed. The TUNEL and flow cytometry results showed that the number of apoptotic cells in the LPS-treated HK-2 cells was significantly increased (Fig. 7A, B). In contrast, ANXA1 (Ac2-26) obviously markedly reduced apoptosis in LPS-treated HK-2 cells (Fig. 7A, B). To further verify whether ANXA1 (Ac2-26) regulates apoptosis, HK-2 cells were induced with Fpr2. The transfection of Fpr2–siRNA markedly reversed the protective effect of ANXA1 (Ac2-26) on apoptosis in LPS-induced HK-2 cells (Fig. 7A, B). In summary, these results suggest that ANXA1 (Ac2-26) inhibits apoptosis via the Fpr2 receptor in CLP-treated HK-2 cells.

### ANXA1 (Ac2-26) improved renal function, survival and cell viability via the Fpr2 receptor in CLP-treated mice and LPS-treated HK-2 cells

**Fig. 8:**
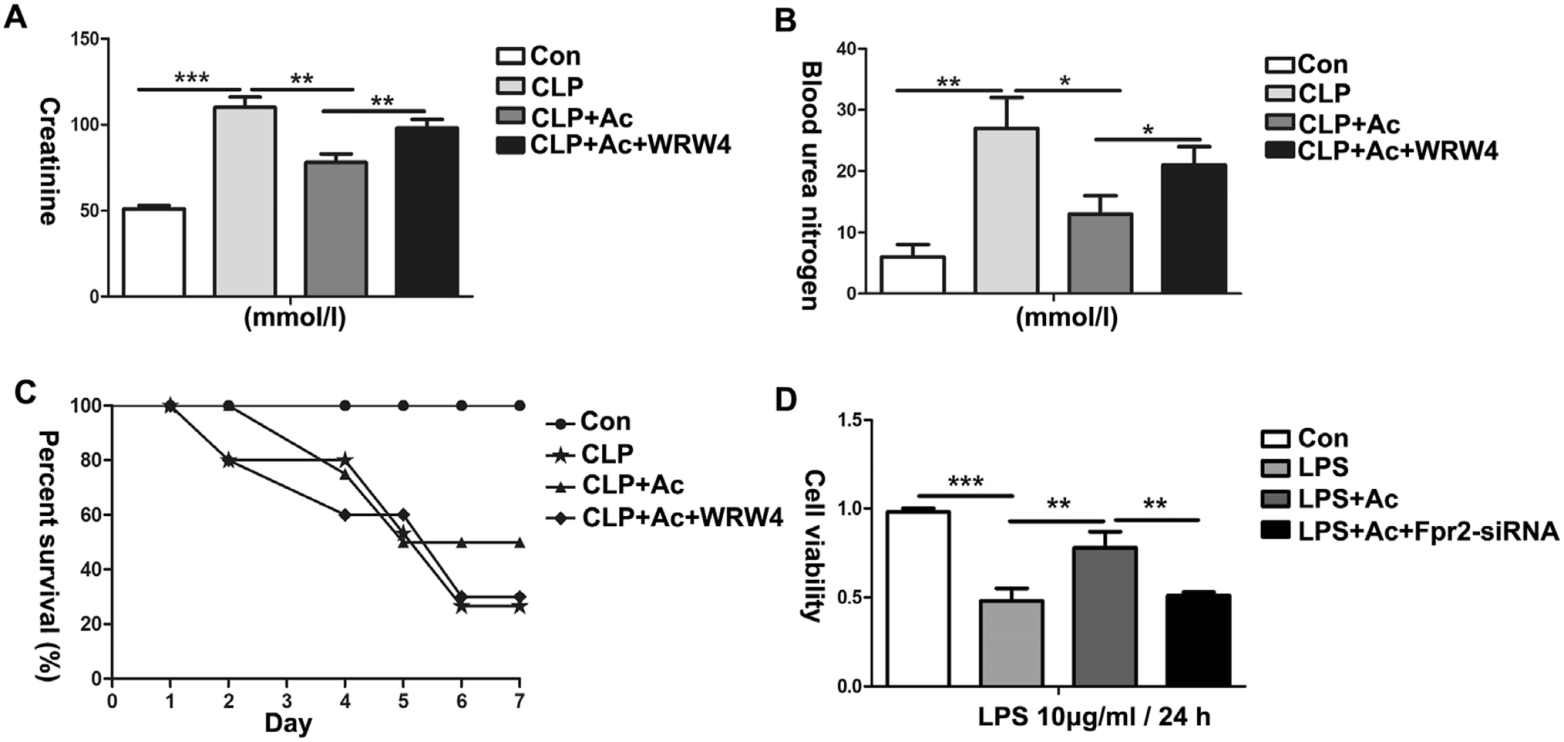
ANXA1 (Ac2-26) protected renal function, survival and cell viability via Fpr2 in CLP-induced mice and LPS-induced HK-2 cells. **A**–**D** Renal function, survival rate and cell viability were detected by ELISA and CCK-8 after CLP or LPS (10 µg/ml) for 24 h. Note: LPS (10 μg/mL), Ac: ANXA1 (Ac2-26) (0.5 μmol/L); Con: control group; LPS: LPS group; LPS + Ac: LPS + Ac2-26 group; LPS + Ac + Frp2-siRNA: LPS + Ac2-26 + Fpr2-siRNA group. The data are represented as the mean ± SEM. The data were independently replicated in triplicate. *P < 0.05, **P < 0.01, ***P < 0.001

To further explore the effect of ANXA1 (Ac2-26) on renal function, the survival rate and cell viability were detected by ELISA and CCK-8 in CLP-induced mice and LPS-induced HK-2 cells, respectively. Our ELISA results showed that the CLP-induced Cre and BUN levels were significantly higher and that the 7-day survival rate of the mice was decreased (Fig. 8A–C). In contrast, ANXA1 (Ac2-26) obviously decreased the levels of Cre and BUN and improved the 7-day survival rate of the mice induced by CLP (Fig. 8A–C). Similarly, the CCK-8 results showed that cell viability was significantly decreased in LPS-treated HK-2 cells (Fig. 8D). ANXA1 (Ac2-26) obviously improved the cell viability induced by LPS in HK-2 cells (Fig. 8D). To further verify whether ANXA1 (Ac2-26) plays the abovementioned regulatory role via the Fpr2 receptor, the transfection of Fpr2-siRNA markedly reversed the protective effects of ANXA1 (Ac2-26) on renal function, survival and cell viability in CLP-induced mice and LPS-induced HK-2 cells (Fig. 8A–D). These results suggest that ANXA1 (Ac2-26) plays the abovementioned regulatory role through the Fpr2 receptor.

## Discussion

This study aimed to verify the hypothesis that ANXA1 (Ac2-26) inhibits inflammation and apoptosis through the Fpr2 receptor to alleviate sepsis-induced AKI and explore its underlying mechanism. In the present study, we found that the levels of ANXA1 (Ac2-26) were downregulated and the levels of proinflammatory cytokines and proapoptotic proteins were increased, which was accompanied by kidney tissue apoptosis in kidneys from CLP-induced mice. Our data show that administration of ANXA1 (Ac2-26) decreased the levels of proinflammatory cytokines and proapoptotic proteins, ameliorated pathological kidney damage, inhibited apoptosis in kidney tissue and HK-2 cells, and improved renal function, the 7-day survival rate of the mice and HK-2 cell viability in CLP-induced mice and LPS-induced HK-2 cells. We also elucidated whether ANXA1 (Ac2-26) plays a regulatory role through the Fpr2 receptor, and the Fpr2 receptor was blocked by administration of WRW4 or Fpr2–siRNA to study its effect on ANXA1 (Ac2-26) in vivo and in vitro. The results showed that the above regulation by ANXA1 (Ac2-26) was reversed by WRW4 and Fpr2–siRNA in CLP-induced mice and LPS-induced HK-2 cells. Mechanistically, ANXA1 (Ac2-26)-elicited effects on SI-AKI largely depend on the negative regulation of inflammation and apoptosis by the Fpr2 receptor.

Increasing evidence shows that an excessive inflammatory response and apoptosis play important roles in the development of sepsis-associated diseases, such as AKI [14, 15]. The inflammatory response induced by LPS through the production and release of a large amount of proinflammatory cytokines, such as TNF-α, IL-2, and IL-6, eventually leads to renal tubular epithelial cell damage and SI-AKI [40]. It has been shown that ANXA1 (Ac2-26) reduces the production of proinflammatory cytokines, such as TNF-α, IL-1β and IL-6, in LPS-induced uveitis [39]. In addition, ANXA1 (Ac2-26) has become an effective anti-inflammatory therapeutic target based on its ability to reduce inflammatory responses in vitro and in vivo and has organ-protective effects [22]. ANXA1 (Ac2-26) is a well-established anti-inflammatory compound that has shown therapeutic potential in a variety of disease models, including lung injury, acute colitis, kidney transplantation and diabetic nephropathy [41]. How does ANXA1 (Ac2-26) regulate inflammatory responses in vivo and in vitro? Further studies revealed that when combined with endogenous anti-inflammatory ligands (such as ANXA1 and resolvin D1) with Fpr2 receptors, downstream molecules that produce anti-inflammatory effects were activated [42]. Researchers found that endogenous inflammatory termination signals could be transmitted by Fpr2 receptor-specific binding to LXA4 [43, 44]. LXA4 belongs to one of the members of Lipoxin, including LXA4, 15-EPI-LXA4, LXB4, and 15-EPI-LXB4. The levels of LXA4 and Fpr2 were significantly elevated in mouse lung tissue in the early stage of sepsis[44]. It has been reported that LXA4 can attenuate the inflammatory response and increase mouse survival in a CLP-induced sepsis model [45]. Some scholars have found that LXA4 attenuates the inflammatory response by downregulating the production of the proinflammatory factors IL-1β, TNF-α, and IL-6 via the Fpr2 receptor in Aspergillus fumigatus keratitis mouse models [46].

Previous studies have found that ANXA1 (Ac2-26) downregulates the level of PI3K/AKT through the Fpr2 receptor, thereby reducing inflammation in CLP-induced mouse myocardial tissue and LPS-induced H9c2 cells [22]. In addition, pinocembrin can inhibit LPS-induced phosphorylation of PI3K/AKT and NF-κB activity, leading to decreased production of inflammatory factors, such as TNF-α and IL-1β and then inhibiting inflammation in BV2 microglial cells [47]. Similarly, we found that ANXA1 (Ac2-26) inhibited the CLP- or LPS-induced production of TNF-α, IL-1β and IL-6, accompanied by the inhibited phosphorylation of PI3K and AKT and downregulated levels of NF-κB in kidney and HK-2 cells (Figs. 2A, B, 5C, D). To demonstrate whether ANXA1 exerts a regulatory effect on inflammation through the Fpr2 receptor, WRW4 and Fpr2-siRNA were administered to block the Fpr2 receptor in vivo and in vitro, respectively. Experimental data show that blockade of the Fpr2 receptor by WRW4 and Fpr2-siRNA reversed the negative regulation of ANXA1 (Ac2-26) on inflammation, accompanied by enhanced phosphorylation of PI3K and AKT and upregulated levels of NF-κB in CLP-induced mice and LPS-induced HK-2 cells (Figs. 2A, B, 5C, D). The phosphorylation of PI3K/AKT levels is closely associated with NF-κB activity, inflammatory factor production and the inflammatory response. Therefore, we speculate that ANXA1 (Ac2-26) may reduce the inflammatory response through the Fpr2 receptor.

Apoptosis is closely related to acute kidney injury in sepsis [13–15]. Zhao et al. found that glycyrrhizic acid alleviates SI-AKI by inhibiting apoptosis in kidney tissue [48]. ANXA1 has been shown to have multiple biological activities, including carcinogenesis, cell proliferation, apoptosis, metastasis, and anti-inflammation [39]. Overexpression of ANXA1 inhibits PI3K/AKT phosphorylation, thereby reducing the activity of caspase-3 and upregulating Bcl-2 expression levels, leading to anti-apoptosis in human bronchial epithelial cells [49]. In addition, Zhang et al. found that ANXA1 (Ac2-26) negatively regulated the PI3K/AKT pathway through LXA4 and subsequently inhibited the activities of caspase-3 and caspase-8 to improve the apoptosis of myocardial tissues and H9c2 cells in vitro and in vivo [22]. It is currently believed that the PI3K/AKT/NF-κB pathway is closely related to apoptosis [50]. In this study, our results demonstrate that treatment with ANXA1 (Ac2-26) markedly downregulated the CLP- or LPS-induced levels of the proapoptotic proteins cleaved caspase-3, cleaved caspase-8, FADD, and Bax, upregulated the level of the antiapoptotic protein Bcl-2, inhibited apoptosis in kidney tissue and HK-2 cells, inhibited the phosphorylation of PI3K and AKT and downregulated the levels of NF-κB in kidney and HK-2 cells (Figs. 3, 4, 6, 7). We again administered WRW4 and Fpr2-siRNA to verify whether ANXA1 (Ac2-26) negatively regulates apoptosis through the Fpr2 receptor. Our data show that blockade of the Fpr2 receptor by WRW4 and Fpr2-siRNA reversed the negative regulation of ANXA1 (Ac2-26) on apoptosis, accompanied by enhanced phosphorylation of PI3K and AKT and upregulated levels of NF-κB in CLP-induced mice and LPS-induced HK-2 cells (Figs. 3, 4, 6, 7). Therefore, the results of this study suggest that ANXA1(Ac2-26) inhibits apoptosis through the Fpr2 receptor. We also found that ANXA1 (Ac2-26) significantly reduced CLP- or LPS-induced pathological kidney injury, improved kidney function, improved the 7-day survival rate, and increased HK-2 cell viability in vivo and in vitro (Figs. 2C, 8). Similarly, administration of WRW4 and Fpr2-siRNA to block the FPR2 receptor reversed the aforementioned protective effect of ANXA1 (Ac2-26) in CLP-induced mice and LPS-induced HK-2 cells (Figs. 2C, 8). Therefore, based on the results of the study, we believe that ANXA1 (Ac2-26) may negatively regulate apoptosis through the Fpr2 receptor.

## Conclusions

**Fig. 9:**
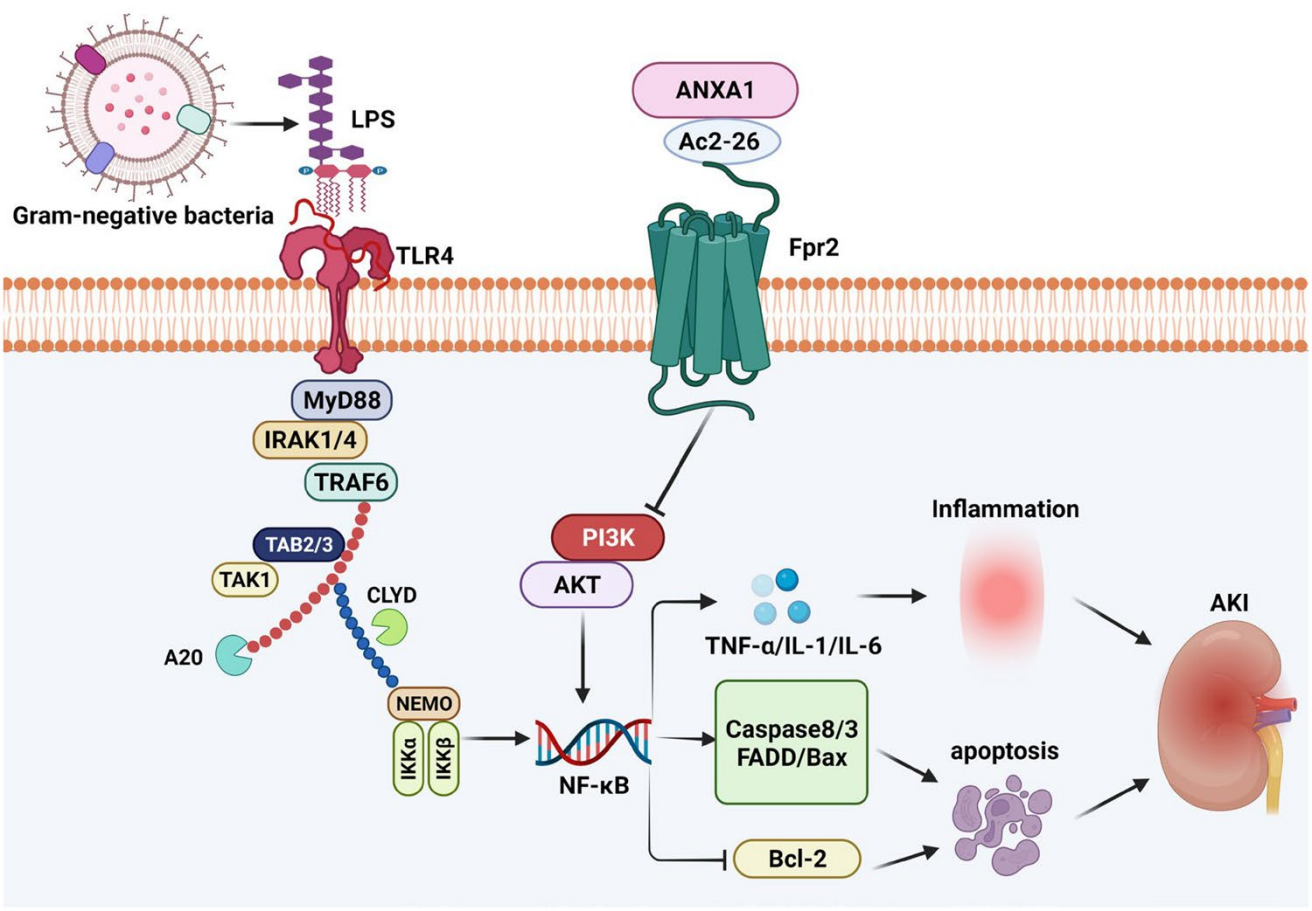
Graphical summary of the potential mechanisms by which ANXA1 (Ac2-26) ameliorates LPS- or CLP-induced AKI via the Fpr2 receptor. Note: the arrow represents promotion; the “tee” head represents inhibition

In summary, the in vivo and in vitro data of our studies revealed that ANXA1 (Ac2-26) attenuated CLP- or LPS-induced inflammation and apoptosis, accompanied by the inhibited phosphorylation of PI3K and AKT and downregulated levels of NF-κB in kidney tissue and HK-2 cells, which might contribute to the protective effect of ANXA1 (Ac2-26) on SI-AKI. The above effects of ANXA1(Ac2-26) were reversed when the Fpr2 receptor was blocked by WRW4 and Fpr2-siRNA. Mechanistically, ANXA1 (Ac2-26) may ameliorate SI-AKI by inhibiting inflammation and apoptosis through the Fpr2 receptor (Fig. 9). These results might aid in the development of new therapeutic strategies for the treatment of SI-AKI. However, further studies are required to fully understand the specific mechanism by which ANXA1 (Ac2-26) contributes to SI-AKI via the Fpr2 receptor.
